# Brr2 plays a role in spliceosomal activation in addition to U4/U6 unwinding

**DOI:** 10.1093/nar/gkv062

**Published:** 2015-02-10

**Authors:** Lingdi Zhang, Xueni Li, Ryan C. Hill, Yan Qiu, Wenzheng Zhang, Kirk C. Hansen, Rui Zhao

**Affiliations:** 1 Department of Biochemistry and Molecular Genetics, University of Colorado School of Medicine, Aurora, CO 80045, USA; 2 College of Bioscience and Bioengineering, Hebei University of Science and Technology, Shijiazhuang 050018, P. R. China

## Abstract

Brr2 is a DExD/H-box RNA helicase that is responsible for U4/U6 unwinding, a critical step in spliceosomal activation. Brr2 is a large protein (∼250 kD) that consists of an N-terminal domain (∼500 residues) with unknown function and two Hel308-like modules that are responsible for RNA unwinding. Here we demonstrate that removal of the entire N-terminal domain is lethal to *Saccharomyces cerevisiae* and deletion of the N-terminal 120 residues leads to splicing defects and severely impaired growth. This N-terminal truncation does not significantly affect Brr2's helicase activity. Brr2-Δ120 can be successfully assembled into the tri-snRNP (albeit at a lower level than the WT Brr2) and the spliceosomal B complex. However, the truncation significantly impairs spliceosomal activation, leading to a dramatic reduction of U5, U6 snRNAs and accumulation of U1 snRNA in the B^act^ complex. The N-terminal domain of Brr2 does not seem to be directly involved in regulating U1/5'ss unwinding. Instead, the N-terminal domain seems to be critical for retaining U5 and U6 snRNPs during/after spliceosomal activation through its interaction with snRNAs and possibly other spliceosomal proteins, revealing a new role of Brr2 in spliceosomal activation in addition to U4/U6 unwinding.

## INTRODUCTION

Pre-mRNA splicing is catalyzed by the spliceosome, a large RNA/protein complex. The spliceosome contains five small nuclear RNAs (U1, U2, U4, U5 and U6 snRNAs) and over 100 protein factors (1,2). The spliceosome typically assembles on each pre-mRNA in a step-wise manner. The spliceosomal assembly, activation and disassembly process involves many adenosine triphosphate (ATP)-dependent conformational rearrangements, which are potentially important for splicing fidelity (3–5). In the first spliceosomal assembly step, the 5′ss is recognized by U1 snRNP, the branch point sequence (BPS) is recognized by branch binding protein (SF1 in mammals) and the polypyrimidine tract is recognized by MUD2 (U2AF^65^ in mammals). The U2 snRNP subsequently joins the spliceosome, followed by addition of the U4/U6.U5 tri-snRNP. Extensive structural rearrangements occur at this stage to activate the spliceosome (6). In this activation process, the base-pairing between the 5′ss and U1 snRNA is disrupted, and the 5′ss interacts with U6 instead, using largely the same nucleotides that base-paired with U1 snRNA. The base-pairing between U4 and U6 is also disrupted, and new interactions between U2 and U6 are formed which are mutually exclusive with those in the original U4/U6 complex. These rearrangements help convert the spliceosome to the catalytically active complex ready for the first step reaction. After the first transesterification reaction, the spliceosome repositions the substrate for the second catalytic reaction. The second reaction is followed by postcatalytic rearrangements that liberate the mature mRNA for export, release the lariat intron to be degraded and the snRNPs to be recycled.

Brr2 is a large (2163 residues in yeast) DExD/H-box protein and a component of the U5 snRNP, tri-snRNP and the spliceosome (7–9). Brr2 is responsible for unwinding U4/U6, a critical step in spliceosomal activation (7–9). Brr2 has an additional role in spliceosomal disassembly possibly by unwinding U2/U6 (10). Mutations in Brr2 have been implicated in the human genetic disorder Retinitis Pigmentosa, potentially by affecting U4/U6 unwinding or 5′ss recognition (11–14). As an integral component of the U5 snRNP, tri-snRNP and spliceosome (15), regulation of Brr2's helicase activity is particularly important to ensure the appropriate timing of spliceosomal activation or disassembly. Both Prp8 and Snu114 (components of the U5 snRNP, tri-snRNP and spliceosome) have been implicated in regulating the activity of Brr2 (6,10,16–18).

Brr2 contains an N-terminal domain followed by two tandem helicase cassettes. Helicase motifs in the first, but not the second helicase cassette, are critical for ATPase activity, U4/U6 unwinding and cell viability (9). Structural analyses reveal that each helicase cassette resembles the DNA helicase Hel308 with an additional fibronectin type three domain at the C-terminus (19–21). The two Hel308-like cassettes (Hel308-I and Hel308-II) are tightly packed against each other (21). The N-terminal helicase cassette can unwind U4/U6 on its own, but with a much lower activity compared to the two cassettes together. Based on the Hel308-DNA structure, the RNA substrate was thought to thread through the enclosure formed by domains 1–5 of Hel308-I, translocate in a 3′ to 5′ direction with the help of the ratchet helix and unwind with the help of the β-hairpin in the second RecA domain of Hel308-I. The C-terminal helicase cassette (Hel308-II) can bind but cannot hydrolyze ATP. The tight packing between the two cassettes may help the N-terminal cassette (Hel308-I) achieve optimal conformation for catalysis. The large size and expanded surface of the C-terminal cassette (Hel308-II) present an opportunity for long distance regulation of the N-terminal cassette (Hel308-I), potentially by multiple protein factors.

The structure and function of the N-terminal domain of Brr2 (∼500 amino acids) are unknown. Here, we demonstrate that removal of the entire N-terminal domain is lethal to *Saccharomyces cerevisiae* and truncation of 120 residues from the N-terminus leads to splicing defects and severely impaired growth. This 120-residue N-terminal truncation does not significantly affect Brr2's helicase activity. The tri-snRNP level in the truncation strain is slightly lower than the WT strain but not sufficient to affect spliceosomal assembly. However, the truncation significantly impairs spliceosomal activation, leading to dramatic reduction of U5, U6 snRNAs and accumulation of U1 snRNA in the B^act^ complex. This truncation does not seem to be directly involved in regulating U1/5'ss unwinding. On the other hand, the N-terminal domain of Brr2 seems to be critical for retaining U5 and U6 snRNPs during/after spliceosomal activation, revealing a new role of Brr2 in spliceosomal activation in addition to its known role in U4/U6 unwinding.

## MATERIALS AND METHODS

### Yeast strains and plasmids

For growth phenotype analyses, various Brr2 N-terminal truncations with a C-terminal polyoma tag were generated by amplifying the pPR150 plasmid (which contains the polyoma-tagged WT *BRR2* (7), all plasmids with their relevant genotypes are listed in Supplementary Table S1) without the deleted region, using polymerase chain reaction (PCR) primers containing a common AatII restriction site for subsequent digestion and religation. The AatII restriction site (GACGTC) introduced three additional residues (the initiator Met plus Asp and Val residues from the AatII site) at the N-terminus of Brr2. The deletion plasmids (pLZ116–118, 121, 133–135, 177) were sequenced to confirm the entire *brr2* sequence is correct and transformed into yAK29 strain (gift from Dr Christine Guthrie, unpublished) for further analyses. All yeast strains with their relevant genotypes and references are listed in Supplementary Table S2.

For other experiments (splicing phenotype, U4/U6 unwinding, tri-snRNP purification, spliceosome assembly and activation, and UV crosslinking), we removed the polyoma tag by NotI digestion in pPR150 (7) and pLZ116 and replaced it with a TAP tag (calmodulin binding peptide-TEV cleavage site-protein A) to generate plasmids pLZ185 (*BRR2* WT) and pLZ186 (*brr2-Δ120*). We transformed these plasmids into yeast strain yAK29, then selected against the *URA3* plasmids on 5-FOA plates to obtain yeast strains yLZ194 and yLZ196 for subsequent experiments.

U1 plasmids (pAK8 = U1-WT and pAK10 = U1-4U) are gifts from Dr Christine Guthrie (22). We generated U1-2A/10A mutation from the U1-WT plasmid using a modified QuikChange protocol (23), and confirmed the mutant through DNA sequencing (pLZ218). Since all U1 plasmids carry a TRP marker, yLZ221 was generated by crossing PRY118 (7) to AXY1413 (Supplementary Table S2) with the goal of disrupting *TRP1* in PRY118. Diploids were sporulated using the standard protocol (24) for tetrad dissection. Haploids that were Trp^−^ Leu^+^ Ura^+^ G418^r^ were screened for *pmr1::kanMX4* using PCR to select haploids that only carry *trp1Δ::kanMX4* to generate yLZ221. The two sets of primers used for PCR (pmr1 forward and Kan reverse; Kan forward and pmr1 reverse) are listed in Supplementary Table S3.

The U1 plasmids (pAK8, pAK10, pLZ218) were co-transformed with pLZ185 or pLZ186 into yLZ221 to obtain yeast strains with the appropriate combination of U1 and Brr2 constructs. The yeast strains were then grown in −Trp/−His media and plated on 5-FOA plates to select against the *pSE360-BRR2* plasmid. These yeast strains with various combinations of U1 and Brr2 constructs were used to evaluate the genetic interaction between Brr2 and U1/5'ss unwinding. All results in these experiments were also confirmed using U1 WT, U1-4U, U1-2A/10A plasmids carrying a LEU marker in the yAK29 strain.

The Act1 reporter plasmid pCG90 (WT *ACT1*) and pCG91 (*act1-10bp*) are gifts from Drs Jonathan Staley and Christine Guthrie (22). The reporter plasmids were transformed into yeast strains yLZ194 (WT *BRR2*) and yLZ196 (*brr2-Δ120*) for further analyses.

### Growth phenotype analyses

**Figure 1.**
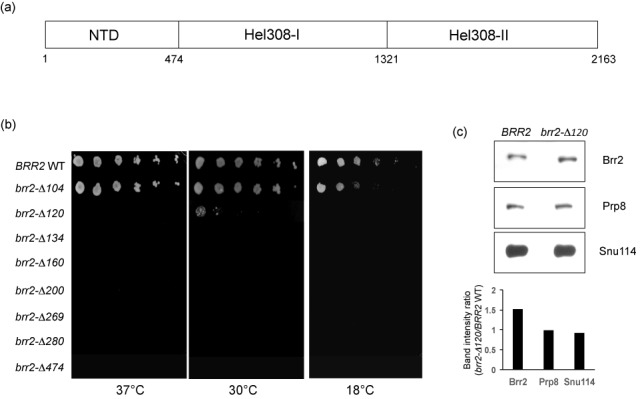
Most of Brr2 N-terminal deletions demonstrate lethal or slow growing phenotypes in yeast. (**a**) A schematic representation of the Brr2 domain structure. (**b**) Most Brr2 N-terminal deletions are lethal or slow growing. (**c**) Polyoma tagged Brr2 was immunoprecipitated from whole cell lysate and various protein levels were evaluated using Western blot analyses. A quantification of the band intensity ratios in *brr2-Δ120* versus *BRR2* WT is also shown. All band intensities in this and other figures are quantified using Quantity One (Bio-rad). *brr2-Δ120* does not significantly affect Brr2 protein level while Prp8 and Snu114 protein levels associated with Brr2 are slightly lower after normalized to the same amount of Brr2 proteins.

For various *brr2* truncations, three independent colonies from the transformation plate were streaked on 5-FOA plates and incubated at 30°C for 4 days. Most truncation constructs are inviable on 5-FOA plates except *brr2-Δ120* and *brr2-Δ104*. For growth phenotype analyses, three colonies per construct were picked from the transformation plate, grown in −His media at 30°C overnight, refreshed in −His media and grown until OD_600_ = 0.8–1, then serially diluted (1:5 dilution from a starting OD_600_ of 0.4) and dotted on 5-FOA plates. The plates were incubated at 30 and 37°C for 4 days and at 18°C for 6 days. All three colonies have the same growth phenotypes and image from one colony is presented in Figure 1b.

For growth phenotype analyses of yeast strains containing a combination of U1 constructs (U1-WT, U1-4U, U1-2A/10A) and *BRR2* WT or *brr2-Δ120* (Figure 6b), yeast cells were grown in −His/−Trp media to OD_600_ ∼0.8–1, dotted onto −His/−Trp plates in a 5× serial dilution (starting from OD_600_ of 0.4) and incubated at 30°C for 4–6 days.

**Figure 2.**
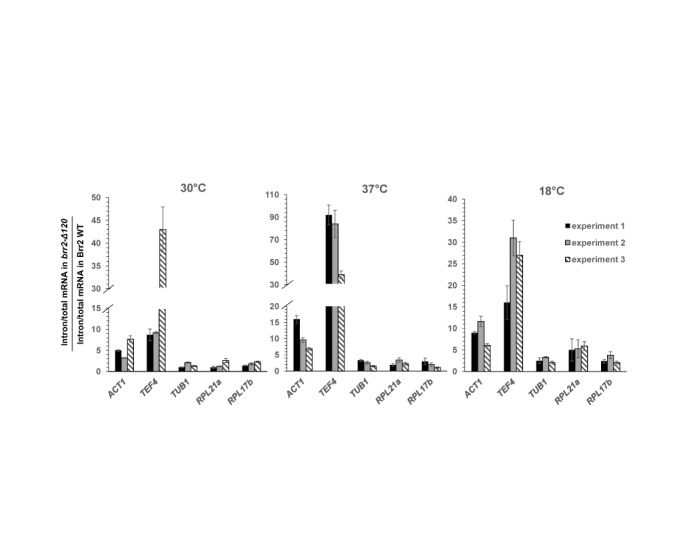
*brr2-Δ120* leads to splicing defects as demonstrated by pre-mRNA accumulation of multiple intron-containing yeast genes at different temperatures measured by real time PCR. Data from three independent experiments are shown and error bars represent standard deviations of three technical replicates in each experiment.

**Figure 3.**
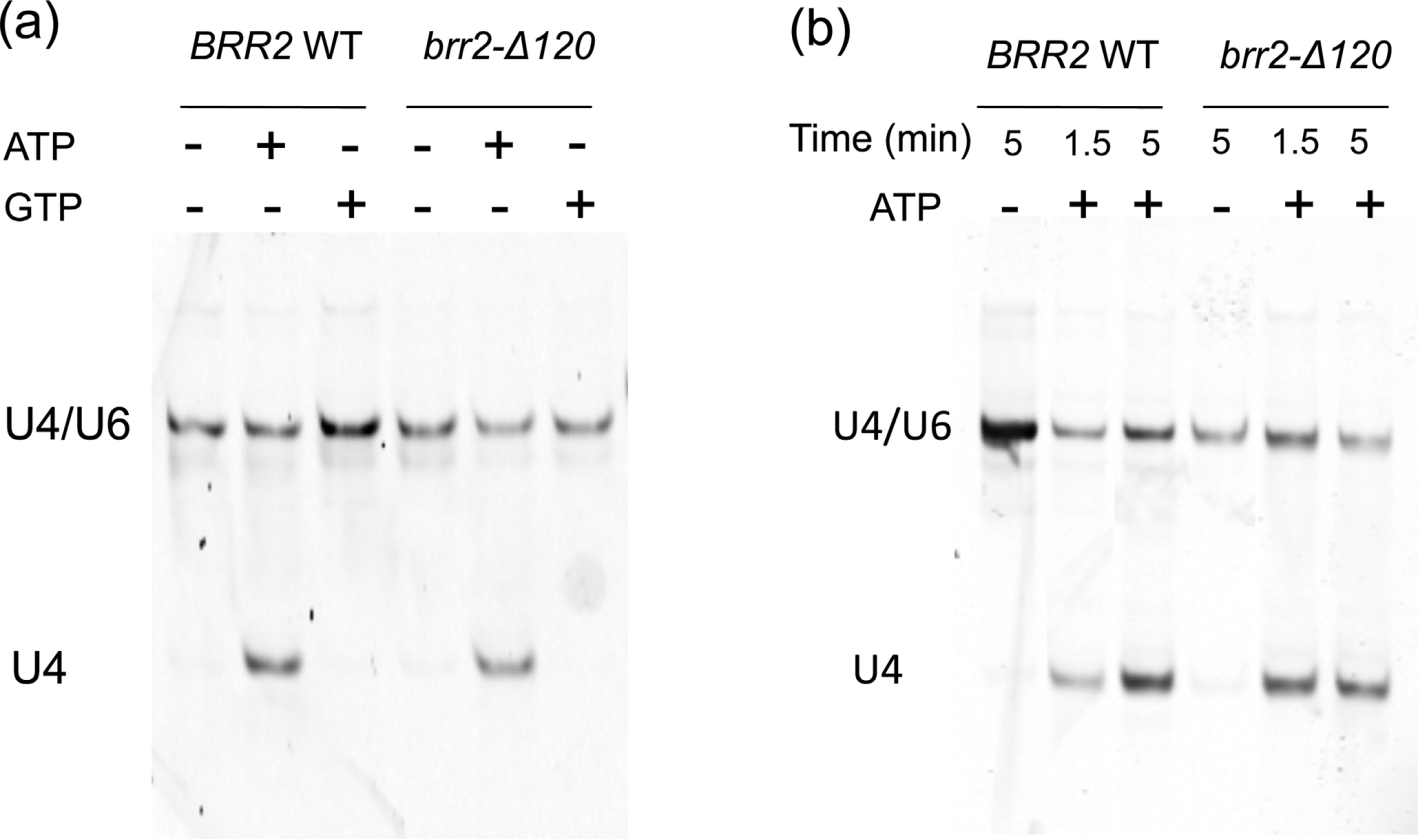
*brr2-Δ120* does not affect the extent of U4/U6 unwinding in tri-snRNP after 1.5–15 min. Affinity purified tri-snRNP using a TAP-tag on Brr2 was either (**a**) incubated with no nucleotide/ATP/GTP for 15 minutes, or (**b**) incubated with ATP for 1.5 and 5 minutes. U4/U6 unwinding is evaluated using solution hybridization with a fluorescence probe that specifically hybridizes to U4 snRNA (which is then analyzed on native polyacrylamide gel and visualized on the Odyssey Infrared Imaging System).

**Figure 4.**
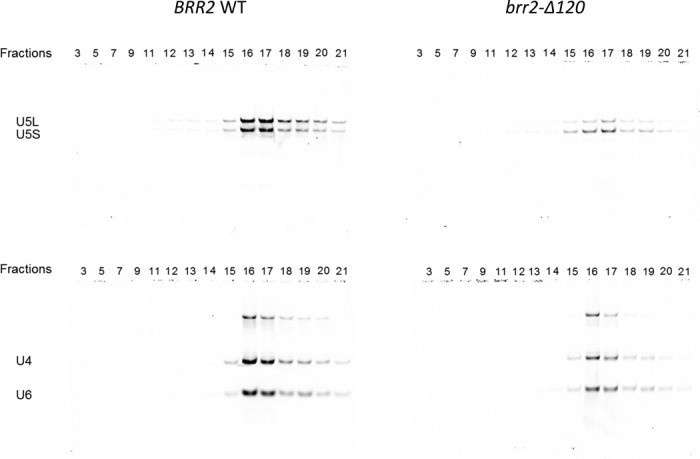
*brr2-Δ120* is able to form tri-snRNP demonstrated on a glycerol gradient. RNA from each fraction is analyzed using solution hybridization with fluorescent probes specific for U5 snRNA (top gels) and for U4 and U6 snRNAs (bottom gels). Solution hybridization samples were analyzed on native polyacrylamide gel and visualized on the Odyssey Infrared Imaging System. The top band on the gel probed with U4 and U6 probes is likely incompletely denatured U4/U6 duplex.

**Figure 5.**
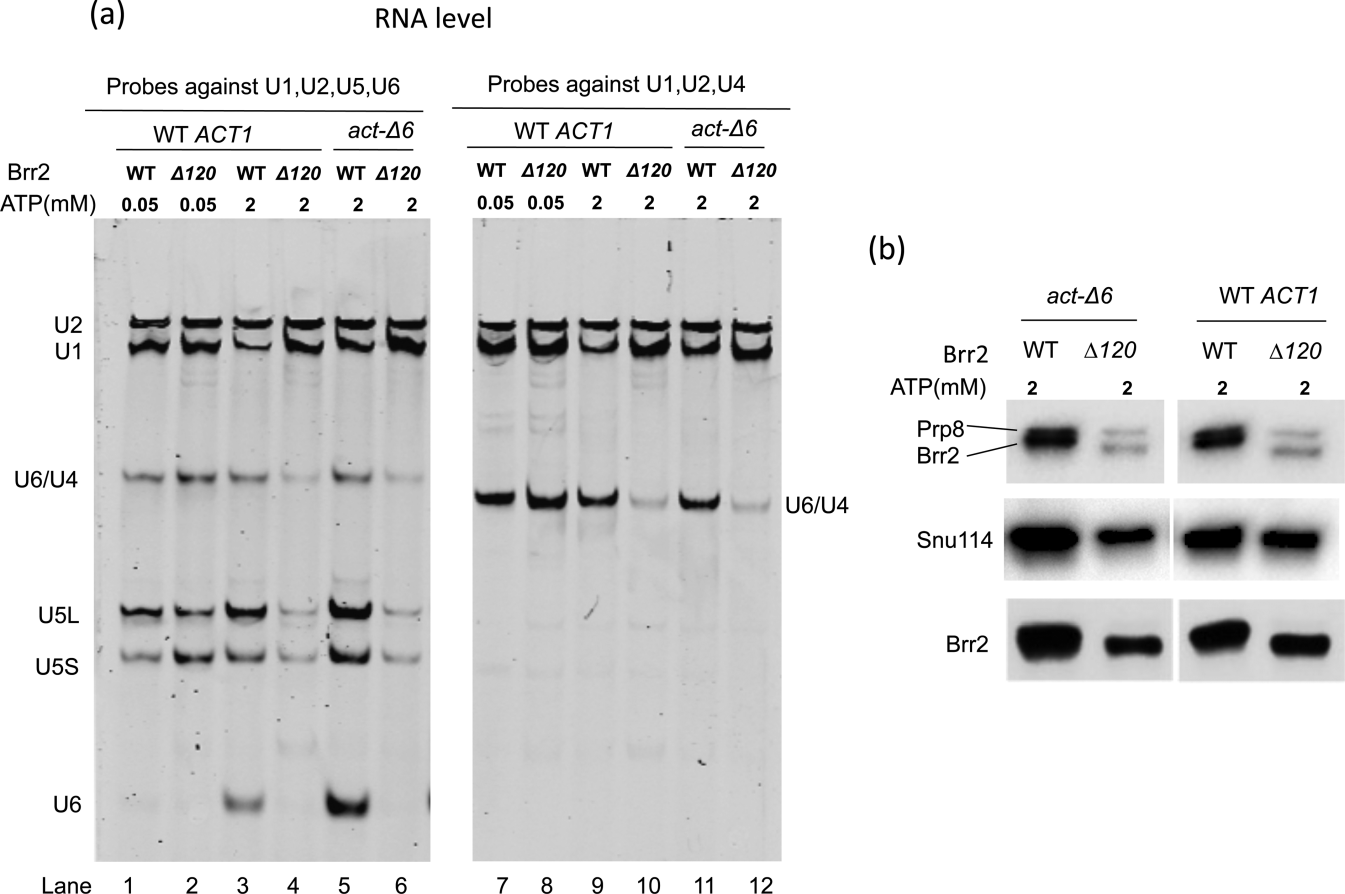
Brr2-Δ120 does not significantly affect spliceosomal assembly, but drastically inhibits spliceosomal activation. (**a**) RNA levels of spliceosomal B complex assembled on WT *ACT1* pre-mRNA with 0.05 mM ATP are similar in WT and *brr2-Δ120* strains (lanes 1, 2, 7, 8). However, spliceosomal B^act^ complex assembled using *brr2-Δ120* extract on *act-Δ6* pre-mRNA in the presence of 2 mM ATP demonstrated significant U1 snRNA accumulation and U4/U5/U6 reduction (lanes 5, 6, 11, 12). Spliceosome assembled on WT *ACT1* pre-mRNA in the presence of 2 mM ATP demonstrate similar U1 snRNA accumulation and U4/U5/U6 reduction (lanes 3, 4, 9, 10). RNA samples were analyzed using solution hybridization followed by electrophoresis on native polyacrylamide gel and visualization on the Odyssey Infrared Imaging System. (**b**) Western blot of proteins in the assembled B^act^ complex demonstrate that there is a significant reduction of Prp8, Brr2 and Snu114 in *brr2-Δ120* compared to the WT. The anti-Prp8 antibody also recognizes Brr2 proteins due to the TAP tag on Brr2.

**Figure 6.**
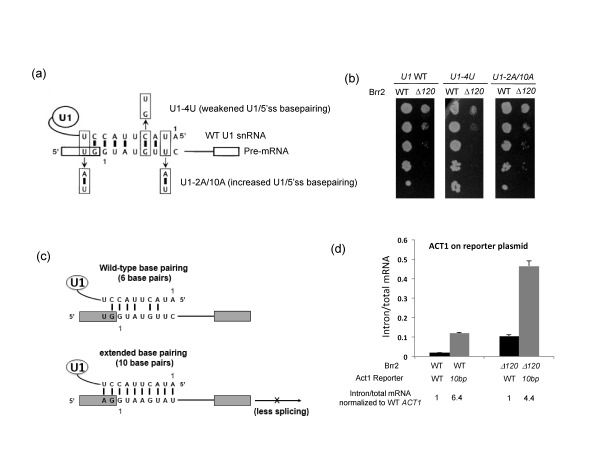
Genetic interaction between Brr2-Δ120 and U1/5'ss unwinding. (**a**) A schematic representation of the U1-4U that weakens the basepairing of the *ACT1*5'ss and U1 interaction and U1-2A/10A mutant that extends the basepairing of the *ACT1*5'ss and U1 snRNA interaction to 10 bp (adapted from (22)). (**b**) There is no significant growth difference at 30°C between *brr2-Δ120* carrying either the U1-4U mutation or the U1-2A/10A mutation and WT U1. Similar observations can also be made at other temperatures (Supplementary Figure S2). (**c**) A schematic representation of the WT *ACT1* and *act1-10bp* reporter which increases the 5'ss and U1 snRNA interaction to 10 bp (adapted from (22)). (**d**) The intron/total mRNA values of *ACT1* or *act1-10* bp on reporter plasmids in either the WT and *brr2-Δ120* strain were evaluated using real time PCR. The ratio of intron/total mRNA values between the *act1-10* bp reporter and *ACT1* reporter is also listed at the bottom of the diagram.

### Real time PCR

We used real time PCR to evaluate the splicing phenotype of several genes (*ACT1, TEF4, TUB1, RPL21a and RPL17b*) in WT and *brr2-Δ120* strains. Total cellular RNAs were extracted from each strain by incubating cell pellet from 5 ml culture with 400 μl RNA lysis buffer (10 mM Tris–HCl (pH8.0), 100 mM NaCl, 1 mM ethylenediaminetetraacetic acid (EDTA), 2% Triton-100, 1% sodium dodecyl sulphate (SDS)), 400 μl phenol/chloroform (PH5.2) and 250 μl autoclaved glass beads. Cells were lysed on an Air Cooling Bullet Blender (Next Advance) at 4°C, centrifuged and RNAs were extracted from the supernatant twice using phenol/chloroform (pH 5.2) and once using chloroform/isopropanol. Then 1/10 volume of 0.3 M NaAC, 0.4 μl GlycoBlue™ (Ambion) and 2.5 volume of ethanol were added to precipitate RNA at −80°C overnight. The RNA was treated with DNase I (Roche), phenol/chloroform (pH5.2) extracted and precipitated, treated by DNase I again, and the reaction was stopped with ethylene glycol tetraacetic acid (EGTA). 500 μg DNaseI-treated RNAs were reverse transcribed into cDNAs using Random Primer Mix and ProtoScript II^®^ Reverse Transcriptase (New England Biolabs). The cDNA was amplified with real time PCR on LightCycler^®^ 480 (Roche) using a set of primers specific for the intron to evaluate the level of intron-containing pre-mRNAs and another set of primers specific for the exon to evaluate the level of total mRNAs. The ratio of intron/total mRNA in *brr2-Δ120* to intron/total mRNA in WT was used to compare the splicing efficiency of each gene in *brr2-Δ120* and WT. Primers for *ACT1, RPL21a and RPL17a* are reported in (25) and primers for *TUB1* and *TEF4* are listed in Supplementary Table S3. Primers for analyzing the splicing phenotype of the Act1 reporter gene in Figure 6D are also listed in Supplementary Table S3. To analyze the splicing phenotype at restrictive temperatures, the yeast strains yLZ194 (WT *BRR2*) and yLZ196 (*brr2-Δ120*) were grown to OD_600_ of 0.6–0.8 at 30°C, then shifted to 37 or 18°C and continued shaking for 3 h before harvesting the cells for RNA extraction.

### Brr2 pull down assay

We cultured yeast cells to OD_600_ of 3–4 and lysed the cells using a SPEX SamplePrep 6870 Freezer Mill (SPEX CertiPrep). We pulled down TAP-tagged Brr2 from the yeast extract using IgG resin in yeast extract buffer (YEB) (40 mM HEPES pH7.9, 200 mM NaCl, 1.5 mM MgCl_2_, 0.01% NP40), which is known to pull down Brr2-containing snRNPs (26). To analyze RNAs associated with Brr2, 35 ml YEB buffer (with protease inhibitors) was added to yeast cell lysate from 2 l of culture. The cell lysate was centrifuged at 18,000 rpm with a Sorvall SS-34 rotor at 4°C for 40 min. The supernatant was then transferred to a Ti-70.1 tube and centrifuged at 42,000 rpm at 4°C for 1 h. Brr2 proteins were pulled down on IgG resin (GE Healthcare) by incubating the resin with yeast extract overnight at 4°C and washing the resin with 50× bed volume of YEB buffer at 4°C. After washing, the IgG resin (with the Brr2-containing tri-snRNP on the resin) was used to perform U4/U6 unwinding assay or evaluate RNAs and proteins associated with Brr2.

To quantify RNAs associated with Brr2, proteins on the IgG resin were digested using proteinase K at 37°C for 30 min. The RNAs associated with Brr2 were extracted by phenol/chloroform and precipitated by ethanol. The isolated RNAs were probed by IRDy700-U4 (IRDye700-AGGTATTCCAAAAATTCCCTAC), IRDy700-U6 (IRDye700-AAAACGAAATAAATCTCTTTG) or IRDy700-U5 (IRDye700-AAGTTCCAAAAAATATGGCAAGC) using solution hybridization (27). More specifically, we incubated the appropriate fluorescence probes and RNAs in 1× Solution Hybridization buffer (50 mM Tris–HCl pH7.5, 150 mM NaCl, 10 mM EDTA) at 70°C for 5 min, then anneal the RNA and probes at 37°C for 15 min. We analyzed the hybridization samples with loading dye added on an 8% native polyacrylamide gel, which was then scanned with the Odyssey Infrared Imaging System (LI-COR).

Co-immunoprecipitation assay using an anti-polyoma antibody is performed as previously described (19).

### U4/U6 unwinding assay

To evaluate U4/U6 unwinding, the Brr2-bound IgG resin was washed with NET50 buffer (50 mM Tris–HCl pH7.4, 50 mM NaCl, 0.05% NP-40) twice, then incubated with 2 mM ATP (or no ATP or 2 mM Guanosine-5′-triphosphate (GTP) as negative controls) in splicing buffer (60 mM potassium phosphate, pH7.5, 2.5 mM MgCl_2_, 3% PEG8000, 8% glycerol, 0.08 mM EDTA, 20 mM KCl, 0.2 mM Dithiothreitol (DTT)) at 11°C for 1.5, 5 and 15 min. Proteinase K was added to digest proteins on the IgG resin at 37°C for 30 min. The remaining RNAs was analyzed using a modified solution hybridization protocol with probe specific for U4 (only incubating at 37°C for 15 min without the pre-heating step at 70°C, other details of the assay are described above) to examine the extent of U4/U6 unwinding (27).

### Tri-snRNP purification

To evaluate tri-snRNP formation, we eluted Brr2-containing snRNPs from the IgG resin using TEV protease cleavage. The sample is applied to a 10–30% glycerol gradient and subjected to ultracentrifugation at 29,000 rpm and 4°C for 24 h in a Beckman SW-41 rotor. RNAs were extracted from each fraction and analyzed by solution hybridization with fluorescently labeled probes specific for U4, U5, U6 snRNAs (27). We have previously shown that fractions 12–13 and 16–17 to be the U5 and tri-snRNP particles, based on sedimentation markers and analyses of the RNA and protein contents in these fractions (28).

### Spliceosomal assembly and activation assay

The spliceosomal B and B^act^ complexes were assembled essentially following the protocol reported by Fabrizio *et al*. (29). The M3-*ACT1* or M3-*act1-Δ6* pre-mRNA (the yeast Act1 pre-mRNA is truncated at 6 nucleotides after the BPS and carries three MS2 binding sites) was first incubated with MBP-MS2 fusion protein then with the splicing extract in the presence of 0.05 mM (for B complex) or 2 mM (for B^act^ complex) ATP. The assembled complex was purified using amylose beads and treated by proteinase K. RNAs in the complex was extracted and analyzed using a modified solution hybridization protocol with fluorescently labeled probes specific to each snRNA (only incubating at 37°C for 15 min without the pre-heating step at 70°C) (27). The spliceosome assembled on M3-ActΔ6 at 0.05 mM ATP contains all (U1, U2, U4, U5, U6) snRNAs, signifying the formation of the B complex. The spliceosome assembled at 2 mM ATP contains U2, U5 and U6 but has significantly reduced levels of U1 and U4, signifying the formation of B^act^.

### Protein identification by tandem mass spectrometry

To analyze proteins associated with Brr2 protein using mass spectrometry, 50 μl of protein samples eluted from the IgG resin using TEV protease cleavage were tube gel polymerized to remove salts as previously described (30). Briefly, samples were added to a solution of ammonium persulfate, bis-acrylamide and Tetramethylethylenediamine (TEMED) to polymerize the samples in an Eppendorf tube. Gel pieces were washed, reduced, alkylated and digested with 5 ng/μl sequencing grade trypsin (Promega) by incubating at 4°C (31) for 30 min and then 37°C overnight using a standard proteolytic digest workflow (32). Tryptic digests were acidified and then brought up to final volume (20 μl). Nanoflow reverse-phase LC-MS/MS was performed as described previously (33) on a LTQ Orbitrap-Velos mass spectrometer (Thermo Fisher Scientific) coupled with an Eksigent nanoLC-2D system. Data acquisition was performed using Xcalibur™ (Version 2.1) software. MS/MS spectra from raw data files were directly loaded into Progenesis^®^ (non-linear dynamic) for unbiased integrated peak area analyses. Concatenated peak lists from Progenesis were searched against *S. cerevisiae* of the SwissProt database using an in-house Mascot™ server (Version 2.2.6, Matrix Science). Mass tolerances were ±15 ppm for MS peaks and ±0.6 Da for MS/MS fragment ions. Result files from MASCOT were loaded back into the Progenesis software for annotation and resulting peptide spectral matches and integrated peak areas were exported for further analysis.

### Evaluate Brr2 and RNA interaction using UV crosslinking

Yeast cells were cultured to an OD_600_ of 2, harvested and re-suspended in phosphate buffered saline buffer. Cells were plated on 20 cm dishes and treated with UV at 254 nm using a Stratalinker 1800UV (Stratagene) at 800mJ/cm^2^ to crosslink endogenous proteins and RNAs in the cell (34). Crosslinked cells were lysed and TAP-tagged Brr2 protein was pulled down by IgG resin in buffer (50 mM Tris–HCl pH7.8, 450 mM NaCl, 1.5 mM MgCl_2_, 0.1% NP-40, 5 mM β-Mercaptoethanol with protease inhibitors). The IgG resin was first washed with the above buffer containing 750 mM NaCl, then with the same buffer containing 450 mM NaCl. Brr2 protein was then cleaved by TEV enzyme and further purified with calmodulin resin. The sample was then subjected to sodium dodecyl sulphate-polyacrylamide gel electrophoresis and proteins transferred to a nitrocellulose membrane. A piece of membrane immediately above the Brr2 protein alone band was excised, digested with proteinase K and extracted RNA was then analyzed using reverse transcription and real time PCR. Real time PCR primers for U1, U2, U4, U5, U6, 5S rRNA were reported previously (28) and primers for 18S rRNA are listed in Supplementary Table S3.

## RESULTS

### The N-terminal domain of Brr2 is essential for yeast growth and viability

To investigate the functional role of the N-terminal domain of Brr2 (Figure 1a), we generated a number of serial deletions from position 104 to 474. Deletions of the first 134, 160, 200, 269, 280 and 474 residues of Brr2 are lethal to yeast (Figure 1b). Deletion of the first 120 residues leads to a slow growing phenotype at 30°C and is inviable at 37 and 18°C, while deletion of the first 104 residues does not have an obvious growth phenotype at 30 and 37°C but grows slightly slower than the WT at 18°C (Figure 1b). We showed that the protein levels of WT and Brr2-Δ120 are similar using Western blot after immunoprecipitating polyoma-tagged Brr2 (Brr2-Δ120 protein level is 1.5-fold of the WT Brr2 in this particular experiment) (Figure 1c). We also ensured that the amount of anti-polyoma antibody we used for immunoprecipitation is not limiting and can pull down more Brr2 proteins than the amount shown in Figure 1c (Supplementary Figure S1a). Prp8 and Snu114 protein levels associated with Brr2 in *brr2-Δ120* are slightly lower than in the WT after normalizing to the same amount of Brr2 proteins (Figure 1c). The *brr2-Δ120* truncation strain was used to further query the function of the N-terminal domain of Brr2 in splicing.

### *brr2-Δ120* leads to splicing defects in yeast

To evaluate the effect of *brr2-Δ120* on pre-mRNA splicing, we examined the levels of pre-mRNA for a number of endogenous genes (*ACT1, TEF4, TUB1, RPL21a, RPL17b*) using real time PCR and primers specific for the exon (to evaluate the level of total mRNA) and intron region (to evaluate the level of pre-mRNA) of each gene. Several of these genes (for example *ACT1* and *TEF4*) displayed significant accumulation of pre-mRNA at 30°C in the *brr2-Δ120* strain compared to the WT, with the (intron/total mRNA in *brr2-Δ120*)/(intron/total mRNA in WT) ratio significantly higher than 1 (Figure 2). Most of these genes also displayed pre-mRNA accumulation in the *brr2-Δ120* strain at 18 and 37°C (slightly enhanced accumulation compared to 30°C) (Figure 2). These results indicate that *brr2-Δ120* leads to splicing defects in yeast.

### *brr2-Δ120* does not significantly affect U4/U6 unwinding

Next we evaluated the effect of *brr2-Δ120* on the ability of Brr2 to unwind U4/U6. The TAP-tagged Brr2 was pulled down using IgG resin under conditions known to pull down Brr2-containing U5 and tri-snRNP (35). The resin was incubated with no nucleotide, ATP or GTP for 15 min. The protein components were digested with proteinase K, and the remaining RNAs were probed by solution hybridization (27) using a U4-specific probe, followed by native gel electrophoresis. Incubation with ATP led to similar U4/U6 unwinding after 15 min in both the WT and *brr2-Δ120* strains, whereas neither the absence of nucleotide nor the presence of GTP lead to obvious unwinding (Figure 3). We also incubated tri-snRNP with ATP for shorter time periods (1.5 and 5 min), both demonstrating similar U4/U6 unwinding between *brr2-Δ120* and WT (Figure 3b). These results suggest that *brr2-Δ120* does not significantly affect the unwinding activity of Brr2 and the extent of U4/U6 unwinding in tri-snRNP is similar to WT after 1.5 min.

### Brr2-Δ120 can be assembled into tri-snRNP

To evaluate whether *brr2-Δ120* affects U5 and tri-snRNP assembly, we pulled down TAP-tagged Brr2 or Brr2-Δ120 from the same amount of cells on IgG resin, and eluted the Brr2-containing complexes by cleaving off the protein A tag using TEV protease. The Brr2-containing complexes were separated on a 10–30% glycerol gradient and RNAs in each fraction were analyzed by solution hybridization (27). We have previously shown that U5 snRNP sediments at fractions 12–13 and tri-snRNP at fractions 16–17 (28). Brr2-Δ120 can be assembled into tri-snRNP at fractions 16–17 judging from the snRNA contents in these fractions, although at a lower level than the WT Brr2 (Figure 4). The C-terminal TAP-tag on Brr2 is likely inaccessible in the U5 snRNP and we did not observe obvious U5 snRNP complexes in fractions 11–12 in either the WT or *brr2-Δ120* samples.

### *brr2-Δ120* can assemble the spliceosomal B complex but is defective in spliceosomal activation

We next evaluated whether *brr2-Δ120* affects spliceosomal assembly and activation. We prepared splicing extracts from both WT and *brr2-Δ120* strains and assembled the B complex on M3-*ACT1* pre-mRNA in the presence of 0.05 mM ATP (28,29). We also assembled the activated spliceosomal B^act^ complex using the M3-*act1-Δ6* pre-mRNA substrate in the presence of 2 mM ATP (28,29). The assembled spliceosomal complexes were purified on amylose beads and the RNA components of the purified spliceosomal complexes were analyzed using solution hybridization (27) with probes specific to each snRNA. There is no significant difference in the B complex formation between the WT and *brr2-Δ120* strains (Figure 5a, lanes 1, 2, 7, 8). However, there is significant U1 snRNA accumulation and reduction of U4/U5/U6 snRNAs (notably the absence of free U6 snRNA) in the B^act^ complex in the *brr2-Δ120* strain compared to the WT *BRR2* strain (Figure 5a, lanes 5, 6, 11, 12). Western blot analyses also revealed that the B^act^ complex contained a significantly reduced amount of U5 and tri-snRNP proteins Brr2, Prp8 and Snu114 (Figure 5b). Spliceosome assembled on WT *ACT1* substrate in the presence of 2 mM ATP (likely composed of a mixture of B^act^ and later complexes) resembles the B^act^ complex assembled on *act1-Δ6*, demonstrating U1 snRNA accumulation and U4/U5/U6 reduction in *brr2-Δ120* (Figure 5a, lanes 3, 4, 9, 10).

### Genetic interactions between *brr2-Δ120* and U1/5'ss unwinding

The accumulation of U1 snRNA in the B^act^ complex in *brr2-Δ120* raises the possibility that the N-terminal domain of Brr2 facilitates U1/5'ss unwinding, and the truncation of the N-terminal domain of Brr2 hinders U1/5'ss unwinding, leading to the accumulation of U1 snRNA during spliceosomal activation. To test this possibility, we transformed U1 WT, U1-4U which weakens U1/5'ss basepairing or U1-2A/10A which increases U1/5'ss basepairing in *ACT1* (Figure 6a) (22) into the WT *BRR2* and *brr2-Δ120* strains. These U1 mutant plasmids have previously been shown to demonstrate a dominant phenotype over the endogenous WT U1 snRNA (22). For example, the U1-4U mutant relieves, while the U1-2A/10A mutant exacerbates, the growth defect of the *prp28-1* mutant (Prp28 is the helicase responsible for U1/5'ss unwinding) (22). If *brr2-Δ120* indeed hinders U1/5'ss unwinding, we would expect that *brr2-Δ120* strain carrying U1-2A/10A grows worse, while *brr2-Δ120* strain carrying U1-4U grows better than *brr2-Δ120* strain carrying WT U1. However, neither scenario is observed in our serial dilution assay (Figure 6b at 30°C and Supplementary Figure S2 at other temperatures), arguing against the hypothesis that the N-terminal domain of Brr2 facilitates U1/5'ss unwinding.

Next we used an alternative approach to evaluate the potential genetic interaction between *brr2-Δ120* and U1/5'ss unwinding. We transformed a reporter plasmid containing either the WT *ACT1* pre-mRNA or mutant *act1* pre-mRNA that would form 10 bp with U1 snRNA (22) (Figure 6c, we will refer to this mutant as *act1-10 bp* for simplicity) into the WT *BRR2* or *brr2-Δ120* strains. We then evaluated the splicing phenotypes of *ACT1* on the reporter plasmid. In the WT *BRR2* strain, the *act1-10bp* mutant has lower splicing efficiency and higher intron-containing pre-mRNA accumulation compared to the WT *ACT1* reporter plasmid (intron/total mRNA of *act1-10bp* : intron/total mRNA of WT *ACT1* = 6.4), as we would have expected (Figure 6d). In the *brr2-Δ120* strain, both WT and mutant Act1 were spliced less efficiently compared to the WT *BRR2* strain. However, the intron/total mRNA ratio of *act1-10bp* is 4.4-fold of the WT *ACT1* in the *brr2-Δ120* strain, slightly less than the 6.4-fold observed in the BRR2 WT strain. This observation also argues against the hypothesis that the N-terminal domain of Brr2 facilitates U1/5'ss unwinding (which predicts that the *act1-10 bp* mutant will be spliced less efficiently in the *brr2-Δ120* strain compared to the *BRR2 WT* strain).

### Brr2-Δ120 has reduced interaction with snRNAs and spliceosomal proteins

**Figure 7.**
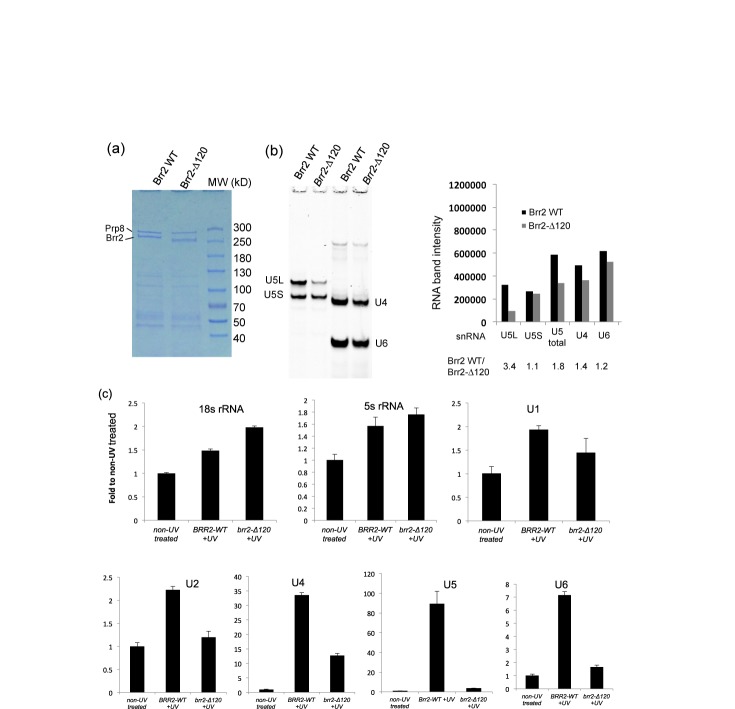
Brr2-Δ120 associates with reduced amount of snRNAs. (**a**) A Coomassie stained SDS PAGE gel showing TAP-tagged Brr2 protein pulled down on IgG resin. (**b**) RNAs associated with Brr2 were analyzed by solution hybridization (followed by electrophoresis on native polyacrylamide gel and visualization on the Odyssey Infrared Imaging System). Brr2-Δ120 associates with less snRNAs than WT Brr2. (**c**) UV crosslinking and real time PCR analyses demonstrated that Brr2-Δ120 has significant less direct association with U2, U4, U5 and U6 snRNAs.

Another major spliceosomal activation defect observed in *brr2-Δ120* is the significant reduction of U5 and U6 snRNAs and proteins in the spliceosomal B^act^ complex (Figure 5). To understand the molecular basis for this defect, we first evaluated RNAs associated with WT and Brr2-Δ120 using solution hybridization after pulling down TAP-tagged Brr2 protein under conditions that primarily pull down U5 and tri-snRNPs (35). Similar levels of Brr2 proteins were pulled down in the WT and *brr2-Δ120* strain judging from Coomassie stained polyacrylamide gel (Figure 7a), consistent with the anti-polyoma immunoprecipitation experiment results (Figure 1c). We also ensured that the amount of IgG resin used for pull down experiments is not limiting and can pull down more Brr2 proteins than the amount shown in Figure 7a (Supplementary Figure S1b). There are small decreases (<2-fold) in the total U5 (1.8-fold), U4 (1.4-fold) and U6 (1.2-fold) snRNAs associated with Brr2-Δ120 (Figure 7b). These results are consistent with our observation that Brr2-Δ120 can be successfully assembled into tri-snRNP but at a lower level than WT (Figure 4). Since this experiment largely pulls down Brr2-associated U5 and tri-snRNPs, it is difficult to evaluate U1 and U2 snRNAs. It is also unclear whether the RNAs evaluated directly or indirectly associate with Brr2.

To overcome the above problems, we next used UV crosslinking in intact cells and real time PCR as a complementary approach to evaluate the interaction between Brr2 and snRNAs. We treated yeast cells with UV irradiation at 254 nm, which crosslinks directly interacting protein and RNA, similar to a CLIP/CRAC experiment (28,34,36). Following cell lysis, TAP-Brr2 proteins were purified in two steps using first IgG and then calmodulin resins and the Brr2 crosslinked snRNAs were evaluated using real time PCR. We observed decreased levels of U2, U4, U5 and U6 snRNAs associated with Brr2-Δ120 compared to the WT Brr2 (Figure 7c). The levels of 18S and 5S rRNA (as negative controls) were not decreased in Brr2-Δ120 compared to the WT Brr2 (Figure 7c). There may be a slight decrease in U1 snRNA as well, although the level of decrease did not reach statistical significance in these experiments (Figure 7c).

**Figure 8.**
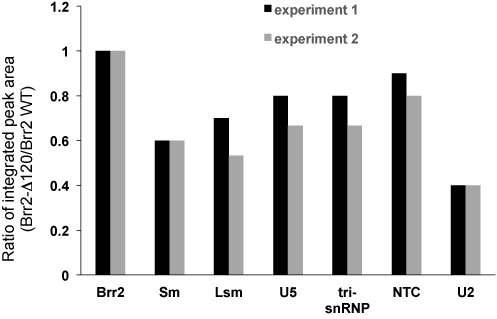
Brr2-Δ120 associates with less U5 snRNP, tri-snRNP, NTC, and U2 snRNP proteins in two independent pull down and mass spectrometry experiments (see Table 1 for raw data). Ratio of averaged integrated peak area (Brr2-Δ120/Brr2 WT) for each group of proteins is shown with this ratio for Brr2 normalized to 1.

We next evaluated spliceosomal proteins associated with Brr2 WT and Brr2-Δ120 proteins using mass spectrometry after pulling down TAP-tagged Brr2 on IgG resin using the same amount of cells and resin (Table 1). The mass spectrometry data carried out in two independent experiments demonstrated again that TAP-Brr2 primarily pulls down U5 and tri-snRNPs under this experimental condition, but there are some NTC/Prp19 complex and U2 snRNP proteins present as well. Brr2 and Brr2-Δ120 protein levels are similar (Brr2-Δ120 protein level is slightly higher than WT Brr2 in experiment 2). Brr2-Δ120 associates with reduced levels of U5 and tri-snRNPs proteins (Figure 8), consistent with the reduced U4/U5/U6 snRNA associations (Figure 7b) and lower amount of tri-snRNP formed (Figure 4). Interestingly, multiple protein components of the U2 snRNP are more significantly reduced, judging by both the spectral counts and integrated peak areas (which typically provide a more accurate quantification of proteins) (Table 1).

**Table 1.** Brr2-Δ120 associates with less U2 snRNP and other spliceosomal proteins

	Pull down experiment 1			Pull down experiment 2		
	Spectrum counts^a^		Integrated peak area	Spectrum counts		Integrated peak area
Proteins	WT	Δ120	Δ120/WT	WT	Δ120	Δ120/WT

**Sm proteins**			**0.6**^b^			**0.9**
SMB	19.5	17	0.7	46.5	42	0.9
SMD1	17	15.5	0.6	41.5	45	1.1
SMD2	17	13.5	0.6	33	27.5	0.8
SMD3	14	11	0.7	41.5	38	0.9
SME	5.5	3	0.5	1	0.5	0.8
SMF	4.5	5.5	0.6	7.5	6	0.7
SMG	4	3.5	0.5	6	6.5	1.1

**Lsm proteins**			**0.7**			**0.8**
LSM2	4	3	0.7	21.5	20.5	0.8
LSM3	2	1	0.6	7.5	7	0.8
LSM4	6	3.5	0.6	16.5	10.5	0.8
LSM5	2	1.5	0.8	4.5	3	0.8
LSM6	7.5	7.5	0.7	5.5	7	1.0
LSM7	2	0.5	0.9	7.5	8.5	1.0
LSM8	6	2.5	0.6	10	10	0.8

**U5 snRNP proteins**			**0.8**			**1.0**
PRP8	99.5	69	0.6	479	444	0.9
BRR2	142.5	142	1.0	453	604.5	1.5
SNU114	46	33.5	0.7	188	174	0.9
DIB1	8	6.5	0.8	28.5	19.5	0.6

**U4/U6.U5 snRNP proteins**			**0.8**			**1.0**
PRP6	51	41	0.7	114.5	115	0.9
PRP31	35	22	0.6	82	71.5	1.0
PRP4	24	17.5	0.7	50	46.5	0.9
PRP3	17.5	12.5	0.7	49.5	55	0.9
SNU66	18	14	0.8	35.5	39	1.4
SNU23	13.5	9	0.7	26.5	28.5	1.0
PRP38	3	2	0.9	21	18	1.0
SPP381	9.5	9.5	0.9	11	14.5	1.1

**NTC/Prp19 complex**			**0.9**			**1.2**
PRP19	2.5	3.5	1.1	29.5	38.5	1.2
CEF1	0	0		1.5	4.5	1.3
CLF1	0	0		2	1.5	2.1^c^
SYF1	1	0	0.6	6.5	6.5	1.0

**U2 snRNP proteins**			**0.4**			**0.6**
RSE1	2	0	0.4^d^	19	10.5	0.6
HSH155	0.5	0	0.3	36	16	0.5
PRP9	0.5	0	0.5	9	4	0.6
PRP21	0	0		3	0	0.6
LEA1	1	0	0.6	7.5	3.5	0.5

^a^Average value of two mass spectrometry runs from the same sample.

^b^Average value for all proteins in this category.

^c^The ratio for Clf1 was not used due to errors in the peak boundary selection.

^d^Although the spectrum count is 0 in Δ120 using the default cutoff in the Progenesis program, integrated peak area can be calculated for the same spectral area corresponding to the WT.

## DISCUSSION

In spite of the clear importance of Brr2 in pre-mRNA splicing, little is known about the structure and function of its large N-terminal domain (∼500 amino acids). We demonstrated that the N-terminal domain of Brr2 is essential for yeast viability and *brr2-Δ120* grows much slower than WT at 30°C and is inviable at 18 and 37°C. We further demonstrated that the Δ120 truncation leads to splicing defects of multiple intron-containing genes in yeast at all three temperatures (Figure 2). Not all yeast intron-containing genes are significantly affected at 30°C, which is not uncommon for mutations in the core spliceosomal proteins (25). For example, Pleiss and colleagues examined the global splicing phenotypes of mutations in 18 core spliceosomal components. They found that each of these mutants affects a subset of the yeast intron-containing genes and the signature of the subset of genes affected is mutant-specific. Inefficient splicing of a subset of genes is likely sufficient to cause growth defects.

Truncating the N-terminal 120 residues in Brr2 does not significantly affect Brr2 protein levels at 30°C (judging from both immunoprecipitation and affinity pull down experiments as shown in Figures 1c and 7a, and Table 1) and the extent of U4/U6 unwinding in tri-snRNP after 1.5 to 15 min (Figure 3). This is consistent with the fact that both the yeast and human Brr2 with the N-terminal domain removed (yeast Brr2 residues 442–2163 and human Brr2 residues 395–2129) are stable and crystal structures of both have been determined (21,37). The human Brr2 with N-terminal truncation has also been shown to be active in U4/U6 unwinding (21). Brr2-Δ120 can be successfully assembled into tri-snRNP, although at a lower level than WT (Figure 4). This modest reduction does not affect assembly of the spliceosomal B complex (the tri-snRNP level is likely not limiting in the spliceosomal assembly assay) (Figure 5a). However, spliceosomal activation is dramatically affected when the first 120 residues of Brr2 is truncated. The B^act^ complex from the *brr2-Δ120* strain shows significant accumulation of U1 snRNA and reduction of U5 and U6 snRNAs (as well as U5 snRNP proteins Brr2, Prp8 and Snu114) (Figure 5). This defect in spliceosomal activation likely leads to the reduced splicing efficiency in *brr2-Δ120*. The mutant phenotype of *brr2-Δ120* (accumulation of U1 snRNA and reduction of U4/U5/U6 snRNAs) has similarities to *prp28-1*, but there are also significant differences between the two mutants. In addition to ineffective disruption of the U1/5'ss interaction, the *prp28-1* mutant is defective in the recruitment of U2 snRNP and CC2 assembly, and the loss of tri-snRNP was thought to be a downstream consequence of unstable CC2 (38). *brr2-Δ120*, on the other hand, does not display obvious assembly defect (no significant loss of U2 or other snRNA in the B complex) and U4/U5/U6 loss is only observed in the activation step (Figure 5a). This suggests that the major defect of *brr2-Δ120* is its inability to retain U5/U6 in the spliceosome upon activation but not during assembly.

The accumulation of U1 snRNA raises the possibility that Brr2 is directly involved in U1/5'ss unwinding. If the N-terminal domain facilitates U1/5'ss unwinding, the N-terminal truncation would lead to the accumulation of U1 snRNA during spliceosomal activation. However, *brr2-Δ120* containing a U1 mutation with longer basepairing does not exacerbate the growth defect of *brr2-Δ120* and neither does *brr2-Δ120* containing a U1 mutation with weaker basepairing suppress the growth defect of *brr2-Δ120* (Figure 6b). In addition, a reporter plasmid carrying a mutant *act1* gene that results in more basepairing with U1 snRNA does not have a worse splicing phenotype than the WT *ACT1* reporter in the *brr2-Δ120* strain (Figure 6d). These observations argue against the hypothesis that the N-terminal domain of Brr2 facilitates U1/5'ss unwinding and suggesting that the U1 accumulation we observed *in vitro* in the B^act^ complex may be a secondary effect (for example as a consequence of the U5 and U6 snRNPs loss in B^act^).

The other major defect observed in spliceosomal activation of *brr2-Δ120* was the dramatic loss of RNAs and protein components of the U5 and U6 snRNPs (Figure 5). It is possible that the N-terminal region of Brr2 is critical for retaining U5 and U6 snRNPs in the spliceosome upon activation. Since Prp28 (the helicase responsible for U1/5'ss unwinding (22)) is a component of the U5 snRNP (26), the loss of U5 snRNP will likely lead to the loss of Prp28 and accumulation of U1 snRNA. In addition, U4/U6 and U1/5'ss unwinding are closely coupled (22,39), U1 unwinding may also be inhibited without the presence of free U6 snRNA (which is absent in B^act^ from *brr2-Δ120*, Figure 5a), leading to U1 snRNA accumulation. Although we cannot completely rule out the possibility that U4/U6 unwinding in the spliceosome is impaired in *brr2-Δ120* (even though the unwinding is similar to WT in isolated tri-snRNP), a defect of U4/U6 unwinding alone is unlikely to lead to the dissociation of U5 and U6 snRNP in the spliceosome. For example, the U4-cs1 mutant is defective in U4/U6 unwinding at the restrictive temperature, but does not lead to U5 and U6 dissociation in the spliceosome (40). Brr2 (especially its NTD) clearly plays a role in retaining U6 and U5 in addition to its function in U4/U6 unwinding, and this retention is critical during or after spliceosomal activation but not before, since B complex is normal in *brr2-Δ120*.

To understand the molecular basis of the loss of U5 and U6 snRNPs in activated spliceosome in *brr2-Δ120*, we evaluated the RNA binding properties of Brr2-Δ120 proteins. Affinity pull-down of the Brr2 protein, under conditions that are known to mostly pull down U5 and tri-snRNP, demonstrated that there is a small reduction (<2-fold) of the amount of U4, U5 and U6 snRNA associated with Brr2-Δ120 (Figure 7b). There is also a reduction of U5 and tri-snRNP proteins associated with Brr2 judging from mass spectrometry analyses (Table 1 and Figure 8). These data are consistent with the lower level of tri-snRNP formation in *brr2-Δ120*, suggesting that the N-terminal region of Brr2 contributes to the interaction between Brr2 and other RNA/proteins in the tri-snRNP. However, this contribution is likely not critical for tri-snRNP assembly, so that Brr2-Δ120 protein can still interact with U4, U5, U6 snRNAs/proteins (likely with a lower affinity) and be successfully assembled into tri-snRNP (albeit at a lower level than WT) (Figure 4) and spliceosomal B complex (Figure 5a).

To further evaluate the association of Brr2 with U1 and U2 snRNA, we carried out UV crosslinking experiments in intact yeast cells followed by stringent purification and real time PCR. The UV crosslinking experiments were carried out under similar conditions as a typical CLIP/CRAC experiment (28,34,36) which is widely used to detect direct protein–RNA associations. These experiments demonstrated that Brr2 directly interacts with U1, U2, U4, U5 and U6 snRNAs *in vivo* (Figure 7c). The direct interaction between Brr2 and U2, U4, U5 and U6 have been reported in a previous CRAC experiment (36). The interaction between Brr2 and U1 snRNP protein Snp1 (directly or indirectly) have been previously observed in yeast two-hybrid and co-immunoprecipitation experiments (41). Our UV crosslinking experiments also demonstrated that Brr2-Δ120 has significantly reduced (2 to 24-fold) direct interaction with U2, U4, U5 and U6 snRNAs compared to WT Brr2 (Figure 7c). The higher extent of reduction observed in crosslinking experiment compared to the pull down experiments (Figure 7b) could be due to the different experimental approaches used. If the reduction of interaction happens to occur on residues critical for crosslinking, it will affect the UV crosslinking experiments much more significantly than the pull down experiments. Additionally, the loss of interaction between Brr2 and RNAs outside of U5 and tri-snRNP may also contribute to the higher extent of reduction observed in UV crosslinking experiments. The reduced interaction between Brr2-Δ120 and U2 snRNA could be an important factor that contributes to the loss of U5 and U6 snRNPs upon spliceosomal activation in *brr2-Δ120*. Consistent with this hypothesis, mass spectrometry analyses demonstrated that Brr2-Δ120 associates with significantly less U2 snRNP proteins compared to WT Brr2 (Table 1, Figure 8). Alternatively, Brr2 can contribute to the retention of U5 and U6 snRNP in activated spliceosome indirectly. The interaction between the N-terminal region of Brr2 and proteins/RNAs in U5/U6 snRNPs may be important for maintaining the U5 and U6 snRNPs in a specific conformation to retain the U5 and U6 snRNPs in the activated spliceosome.The reduced interaction between Brr2-Δ120 and NTC may also contribute to the loss of U5 and U6 snRNPs upon spliceosomal activation in *brr2-Δ120*.

In summary, our results demonstrate an important role of the first 120 residues in the N-terminal domain of Brr2 in spliceosomal activation in addition to the function of Brr2 in U4/U6 unwinding. This region of Brr2 is not critical for U4/U6 unwinding, tri-snRNP formation and spliceosomal assembly. However, this region of Brr2 interacts with U2, U5 and U6 snRNAs (and possibly with other spliceosomal proteins), and these interactions are likely critical for retaining the U5 and U6 snRNPs during/after spliceosomal activation.

## SUPPLEMENTARY DATA

Supplementary Data are available at NAR Online.

SUPPLEMENTARY DATA
