# Supplementary material for: Brr2 plays a role in spliceosomal activation in addition to U4/U6 unwinding
Source: Nucleic Acids Res. 2015 Feb 10;43(6):3286–97. doi: 10.1093/nar/gkv062 (PMC4381053; doi:10.1093/nar/gkv062)
Supplement: SUPPLEMENTARY DATA [file supp_43_6_3286__index.html]

Brr2 plays a role in spliceosomal activation in addition to U4/U6 unwinding — Brr2 plays a role in spliceosomal activation in addition to U4/U6 unwinding — SUPPLEMENTARY DATA 

# Brr2 plays a role in spliceosomal activation in addition to U4/U6 unwinding

## SUPPLEMENTARY DATA

**Files in this Data Supplement:**

- SUPPLEMENTARY DATA
