# Supplementary material for: Brr2 plays a role in spliceosomal activation in addition to U4/U6 unwinding
Source: Nucleic Acids Res. 2015 Feb 10;43(6):3286–97. doi: 10.1093/nar/gkv062 (PMC4381053; doi:10.1093/nar/gkv062)
Supplement: SUPPLEMENTARY DATA [file supp_gkv062_nar-03064-f-2014-File003.pdf]

Supplementary Table 1

Plasmids used in this study

Plasmid	Genotype	Source
pPR150	*ARS CEN HIS3 BRR2-Pya*	Raghunathan and Guthrie, 1998
pLZ133*	*ARS CEN HIS3 brr2-Δ(1-104)-Pya*	This study
pLZ116*	*ARS CEN HIS3 brr2-Δ(1-120)-Pya*	This study
pLZ134*	*ARS CEN HIS3 brr2-Δ(1-134)-Pya*	This study
pLZ135*	*ARS CEN HIS3 brr2-Δ(1-160)-Pya*	This study
pLZ121*	*ARS CEN HIS3 brr2-Δ(1-200)-Pya*	This study
pLZ118*	*ARS CEN HIS3 brr2-Δ(1-269)-Pya*	This study
pLZ117*	*ARS CEN HIS3 brr2-Δ(1-280)-Pya*	This study
pLZ177*	*ARS CEN HIS3 brr2-Δ(1-474)-Pya*	This study
pLZ185	*ARS CEN HIS3 BRR2-TAP*	This study
pLZ186*	*ARS CEN HIS3 brr2-Δ(1-120)-TAP*	This study
pAK8	*ARS CEN TRP1 SNR19*	Siliciano and Guthrie, 1988
pAK10	*ARS CEN TRP1 SNR19-4U*	Siliciano and Guthrie, 1988
pLZ218	*ARS CEN TRP1 SNR19-2A10A*	This study
pCG90	*ARS CEN URA3 ACT1*	Staley and Guthrie, 1999
pCG91	*ARS CEN URA3 act1-10bp*	Staley and Guthrie, 1999
pSE360-BRR2	*ARS CEN URA3 BRR2*	Raghunathan and Guthrie, 1998

* All Brr2 N-terminal trunctionas contain three additional residues (the initiator Met followed by Asp and Val) due to the AatII restriction site used to generate these trunctaions.

Supplementary Table 2

S. cerevisiae strains used in this study

Strains	Relevant Genotype	Source
yAK29	*MAT***a** *his3Δ, ura3Δ, lys2Δ, leu2Δ, brr2::KAN* (pBRR2/URA3)	from Dr. Christine Guthrie, unpublished
yLZ194	*MAT***a** *his3Δ, ura3Δ, lys2Δ, leu2Δ, brr2::KAN* (pLZ185)	This study
yLZ196	*MAT***a** *his3Δ, ura3Δ, lys2Δ, leu2Δ, brr2::KAN* (pLZ186)	This study
PRY118	*MAT***a** *brr2::LEU2 ade2 lys2 his3 ura3 leu2* (pSE360-BRR2)	Raghunathan, 1998
AXY1413	*MATα pmr1::kanMX4 trp1Δ::kanMX4 his3Δ1 leu2Δ0 ura3Δ0*	from Xiuxiang An and Dr. Mingxia Huang, unpublished
yLZ221	*MATα brr2::LEU2 trp1Δ::kanMX4 his3 ura3 leu2* (pSE360-BRR2)	This study

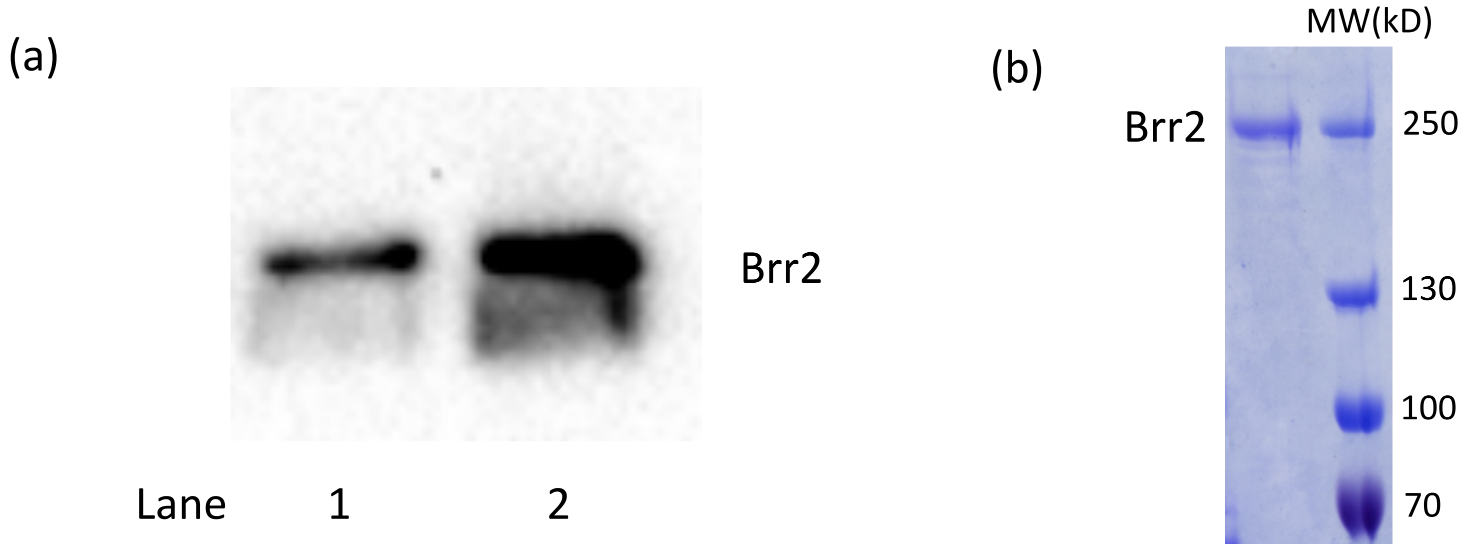

Fig. S1. The amount of anti-polyoma antibody (a) or IgG resin (b) used in immunoprecipitation or pull down experiments in Fig. 1c and 7a have not reached their full binding capacity and are not limiting. (a) Different amounts of yeast extract from the yAK29 (pPR150) strain were pulled down with anti-polyoma antibody and probed with an anti-Brr2 antibody on a Western blot. The amount of anti-polyoma antibody used is the same as Fig. 1c. The amount of yeast extract in Lane 1 is the same as Fig. 1c and that in lane 2 is 9-fold of the amount used in Fig. 1c. (2) A Coomassie stained polyacrylamide gel showing that IgG resin (half of the amount used in Fig. 7a) can pull down much more Brr2 protein from a yeast strain carrying BRR2 under an over-expressing promoter compared to Fig. 7a.

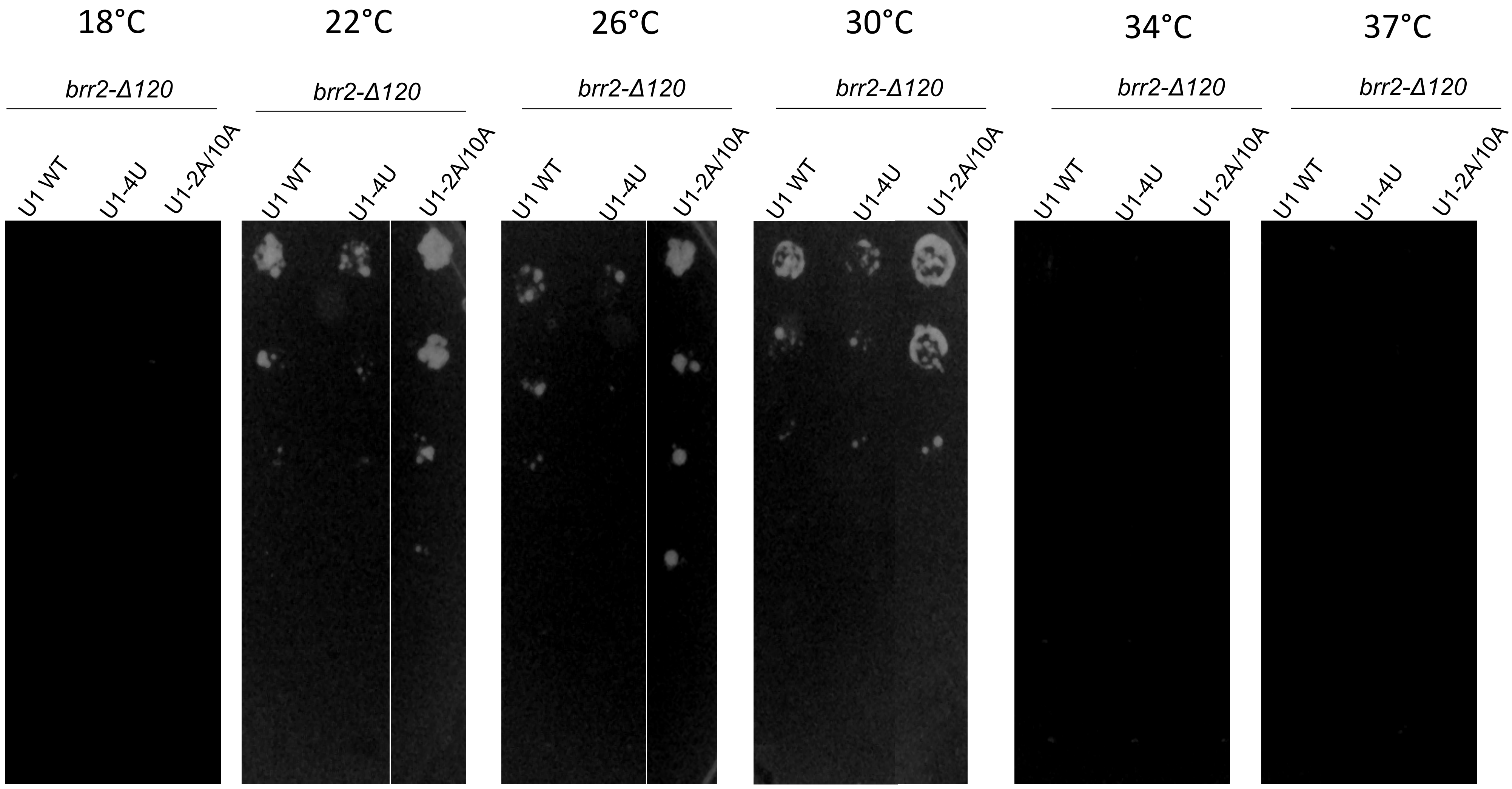

Fig. S2. Growth phenotypes of yeast strains carrying either WT *BRR2* or *brr2-D120* in combination with WT U1, U1-4U, or U1-2A/10A. The growth phenotypes at 22 and 26°C are in general similar to that of 30°C. All *brr2-D120* strains, regardless of the specific U1 plasmids it also carries, are lethal at 18, 34, and 37°C, as we would have expected.
